# Circulating endocannabinoid levels in pregnant women with gestational diabetes mellitus: a case–control study

**DOI:** 10.1186/s12902-022-01182-5

**Published:** 2022-11-03

**Authors:** Hadi Bazyar, Khadijeh Nasiri, Parisa Ghanbari, Elahe Mohammadi, Neda Lotfi Yagin, Maryam Khazdouz, Vahideh Aghamohammadi, Shafagh Ali Asgarzadeh

**Affiliations:** 1 Sirjan School of Medical Sciences, Sirjan, Iran; 2 Student Research Committee, Ahvaz Jundishapur University of Medical Sciences, Ahvaz, Iran; 3 Department of Nursing, Khalkhal University of Medical Sciences, Khalkhal, Iran; 4 Student Research Committee, Ahvaz Jundishapur University of Medical Sciences, Ahvaz, Iran; 5 Department of Nutrition, Khalkhal University of Medical Sciences, Khalkhal, Iran; 6 Endocrine Research Center, Tabriz University of Medical Sciences, Tabriz, Iran; 7 Growth and Development Research Center, Tehran University of Medical Sciences, Tehran, Iran; 8 Department of Internal Medicine, School of Medicine, Imam Khomeini Hospital, Ardabil University of Medical Sciences, Ardabil, Iran

**Keywords:** Anandamide, 2-Arachidonoylglycerol, Endocannabinoids, Gestational Diabetes Mellitus

## Abstract

**Background:**

The role of the Endocannabinoids (ECs) in insulin resistance, and their association with visceral obesity and metabolic profile have been studied extensively. Since the association between ECs and metabolic factors in Gestational Diabetes Mellitus (GDM) are not clear, we aimed to evaluate the levels of N-Arachidonoylethanolamide (AEA) and 2-Arachidonoylglycerol (2-AG) and their association with C-reactive protein (CRP), glycemic indices, blood pressure, and anthropometric indices in pregnant women with GDM.

**Methods:**

The present case–control study was conducted among 96 singleton pregnant women aged 18–40 years, including 48 healthy pregnant women (control group) and 48 women with a positive diagnosis of GDM (case group). Odds Ratios (ORs) and 95% Confidence Intervals (CIs) for GDM were checked according to endocannabinoids and anthropometric indices using Multivariable Logistic Regression.

**Results:**

AEA was significantly associated with increased risk of GDM in models 1, 2 and 3 (OR = 1.22, 95% CI: 1.06–1.41; OR = 1.54, 95% CI: 1.19–1.97; OR = 1.46, 95% CI:1.11–1.91). A positive but no significant association was found for AEA in model 4 (OR = 1.38,95% CI: 0.99–1.92). Similar to AEA, 2-AG was also positively associated with the likelihood of GDM in Models 1, 2, and 3 but the association attenuated to null in model 4 (OR = 1.25; 95% CI: 0.94- 1.65).

**Conclusions:**

Our findings showed that levels of ECs were significantly higher in pregnant women with GDM compared to healthy ones. Also, ECs levels were associated with the likelihood of GDM, independent of BMI and weight gain.

## Introduction

Gestational Diabetes Mellitus (GDM) is the state of the glucose tolerance impairment which is diagnosed during the second and third trimesters of pregnancy. GDM is a growing health problem worldwide and is one of the most common complications of pregnancy. Approximately 14% of the pregnancies are affected by GDM throughout the world yearly [1, 2]. The prevalence of GDM is estimated to be around 11.5% in Asia [3] and its prevalence varies widely from 1.3% to 18.6% in Iran [4]. Obesity, history of GDM, and family history of diabetes are considered as the major risk factors of GDM [5, 6]. Pregnant women with GDM are more likely to develop cesarean section, preeclampsia, and hypertension. Moreover, these women have a significantly higher risk of developing type 2 diabetes mellitus (T2DM), metabolic syndrome, cardiovascular diseases, and depression in later years of their life [7, 8].

Endocannabinoids (ECs) are known as endogenous lipid mediators and are ligands of specific G protein-coupled receptors. Unlike neurotransmitters which are usually stored in vesicles before release, endocannabinoids are synthesized from membrane phospholipid fatty acids based on demand and act as autocrine or paracrine mediators. 2-Arachidonoylglycerol (2-AG) and Anandamide or N-arachidonoylethanolamine (AEA), are derivatives of arachidonic acid, and are the most studied endocannabinoids [9, 10]. Endocannabinoids, their synthesizing and metabolizing enzymes, and their receptors constitute the endocannabinoid system (ECS). It has been revealed that the overactivation of cannabinoid receptors type 1 (CB1) has a significant role in lipogenesis, hepatic steatosis, obesity, and insulin resistance [11–13]. The CB1 receptor is found in CNS and in peripheral organs which control metabolism and activates anabolic pathways in favor of energy storage [14]. The proposed role of the ECS in diabetes pathogenesis has been documented by increased concentrations of ECs in diabetic patients. According to Matias et al., the circulation levels of 2-AG and AEA were higher in T2DM patients compared to healthy volunteers [15]. Although the pancreatic β-cell dysfunction and insulin resistance are the main metabolic alterations in GDM [16], there is still no agreement in GDM pathophysiology. Therefore, complete understanding of the pathophysiology of GDM might help to develop prevention and treatment methods.

Given the putative role of the ECs in insulin resistance, and since the association between ECs and metabolic factors in GDM are not clear, we aimed to evaluate the levels of 2-AG and AEA and their association with C-reactive protein (CRP), glycemic indices, blood pressure, and anthropometric indices in pregnant women with GDM.

## Methods and materials

### Participants

This case–control study was carried out among 96 pregnant women (carrying only one baby) aged 18–40 years, including 48 healthy pregnant women (control group) and 48 women with a positive diagnosis of GDM (case group). The study participants were selected by random sampling from the government Obstetrics and Gynecology Clinics in Ardabil city, Iran(From September 2021 to December 2021).. The sample size was estimated based on the AEA level in T2DM patients attained from the study by Van Eyk et al. using the following formula: N = [ (z_1-α/2_)^2^ × sd^2^]/d^2^ (α = 0.05, sd = 0.41, and d = 0.1312) and 39 subjects were calculated for each group [17]. Finally, regarding the 20% withdrawal rate, 48 subjects were determined for each group. The GDM screening was done between the 24th and 28th weeks of the gestation using a 100 g Oral Glucose Tolerance Test (OGTT). Pregnant women who met the American Diabetes Association (ADA) criteria such as fasting ≥ 95 mg/dl, 1-h ≥ 180 mg/dl, 2-h ≥ 155 mg/dl, were diagnosed as having GDM (any two values equal or above-established thresholds) [18, 19]. Controls were the pregnant women whose OGTT results were in the normal range at the 24th and 28th weeks of gestation. A history of gestational diabetes or diabetes (prepregnancy), having other disorders (such as cardiovascular, liver, and renal disorders), adherence to specific diets, multiple pregnancies, and unwillingness to participate in the study, were exclusion criteria. The participants were fully informed about the study's protocol and they were requested to sign the written informed consent. This study was approved by the Ethics Committee of the Khalkhal University of Medical Science (IR.KHALUMS.REC. 1398.006).

### Measurements

Demographic and physical activity data were acquired through a questionnaire. To assess the participants’ physical activity levels, the short form of the International Physical Activity Questionnaire (IPAQ) was applied [20, 21]. The participants’ food intake was obtained using 24-h recall questionnaires (2 weekdays and 1 weekend day) through the interview with trained dietitians. Macronutrient intakes and total energy intake were then computed using Nutritionist IV software (the Hearst Corporation, San Bruno, CA) as modified for Iranian foods.

Body weight was measured using a Seca scale with an accuracy of 100 gr. Stature was evaluated in standing position without shoes using a Seca stadiometer with an accuracy of 0.5 cm. Then, body mass index (BMI) was computed as the body weight (kg) divided by the square of height (m^2^). The systolic blood pressure (SBP) and diastolic blood pressure (DBP) were determined by a mercury sphygmomanometer (Samsung, Japan) after 5 min resting on a chair in the right arm. For biochemical analyses, 5 cc blood samples were collected from each participant after 10–12 h fasting. To measure the plasma 2-AG and AEA, the blood in EDTA-coated tubes was centrifuged at 1500 g at 4 °C for 15 min and was then stored at -80 °C. ELISA kits (ZellBio, GmbH, Veltinerweg, Germany) were applied to evaluate the plasma levels of 2-AG and AEA. Fasting blood glucose (FBS) was evaluated by the enzymatic method with kits from Pars-Azmoon (Tehran, Iran) with a sensitivity of 5 mg/dL and an internal measurement degree of 1.28. CRP levels were assessed by the human CRP ELISA kit from Pars-Azmoon (Tehran, Iran) with a sensitivity of 2 mg/L. An automated high-performance liquid chromatography (HPLC) exchange ion method (DS5 England) was used to measure HbA1C. Ion-exchange HPLC separates hemoglobin species based on charge differences between HbA1c and other hemoglobins [22]. The chemiluminescent immunoassay method was applied to assess the insulin levels (LIAISON analyzer (310,360) Diasorin S.P.A, Vercelli, Italy). In this assay, a monoclonal antibody is coated on the surface of the test plate and another antibody labeled with CL is used as the indicator. In the presence of insulin, the reacted complex produces chemiluminescence and its intensity is correlated with the insulin concentration [23]. Then, Homeostasis Model Assessment—Insulin Resistance (HOMA-IR) was computed using the following formula: Fasting Glucose(mg/dl) × Fasting Insulin (μu/ml)/405.

### Statistical analysis

All statistical analyses were done using IBM SPSS Statistics software version 24 (IBM SPSS Statistics, Armonk, USA). The normality of the variables was assessed using the Kolmogorov–Smirnov test. The differences in variables between the study groups were tested using an independent sample t-test and Chi-square test for parametric variables and categorical variables, respectively. Linear regression analysis was used in 4 models (Model 1: unadjusted, model 2: adjusted for energy, carbohydrate, protein, and fat, model 3: adjusted for energy, carbohydrate, protein, fat, weight gain, and BMI, model 4: adjusted for energy, carbohydrate, protein, and fat, weight gain, BMI, age, education, job, physical activity level, sex of infant, family history of type 2 diabetes and gestational diabetes, number of pregnancies, and weeks of pregnancy). Also, Odds ratios (95% CI) for gestational diabetes was checked using multivariable logistic regression according to the endocannabinoids (AEA and 2-AG) and anthropometric indices (weight, weight gain, and BMI) in 4 models as stated above. *P* < 0.05 was considered as statistically significant.

## Results

The participants’ characteristics have been presented in Table 1. GDM group had significantly higher weight gain (*P* < 0.001), body weight (*P* = 0.004), and BMI (*P* < 0.001) in comparison with the control group. Moreover, a significant difference was observed between the study groups in terms of the family history of the GDM (*P* = 0.01). Significant differences were observed regarding the biochemical variables and dietary intake, except for protein (*P* = 0.18) and fat (*P* = 0.19) between the study groups (Table 2). The relationship between endocannabinoids (AEA and 2-AG) and FBS, HbA1c, insulin, HOMA-IR, CRP, SBP, and DBP (dependent variables) in the control group has shown in Table 3. In all unadjusted and adjusted models, a positive significant association was observed between the 2-AG and Insulin and HOMA-IR (*P* < 0.05). No significant association was detected between AEA and dependent variables (*P* > 0.05). As shown in Table 4, in models 1 and 2, a positive significant association was identified between 2-AG and AEA with glycemic indices including FBS, HbA1C, insulin blood levels, and HOMA-IR (*P* < 0.05), but these significant associations disappeared in models 3 and 4 in GDM group. Moreover, in model 4, there was a positive significant association between 2-AG and DBP (*P* = 0.02) (Table 4).

**Table 1:** The characteristics of the subjects in the control and GDM groups

Variables	Control group (*n* = 48)	GDM group (*n* = 48)	*P- value*
Age (y)	30.16 ± 1.99	30.87 ± 3.39	0.21^*^
Weight (kg)	69.46 ± 3.56	72.06 ± 5.02	0.004*
Height (cm)	165.68 ± 3.30	165.22 ± 3.24	0.49*
BMI (kg/m^2^)	25.30 ± 1.18	26.37 ± 1.28	< 0.001*
Weight gain (kg)	8.83 ± 1.32	9.95 ± 1.27	< 0.001*
Week of Pregnancy	26.73 ± 1.36	27.08 ± 1.09	0.16*
Education (N) (%)^a^			
High school diploma and sub-diploma	9 (69.2)	4 (30.8)	0.47**
Associate	18 (50)	18 (50)	
Bachelor	14 (43.8)	18 (56.3)	
Master and Ph.D	7 (46.7)	8 (53.3)	
Job (N) (%)			
Official	14 (40)	21 (60)	0.22**
Non-official	19 (61.3)	12 (38.7)	
Housewife	15 (50)	15 (50)	
Family history of GDM (N) (%)			
Yes	0 (0)	6 (100)	0.01**
No	48 (53.3)	42 (46.7)	
Family history of type 2 diabetes mellitus (N) (%)			
Yes	6 (31.6)	13 (68.4)	0.07**
No	42 (54.5)	35 (45.5)	
Number of pregnancies (N) (%)			
First	14 (50)	14 (50)	0.87**
Second	23 (52.3)	21 (47.7)	
Third	11 (45.8)	13 (54.2)	
Levels of physical activity (N) (%)			
Moderate	13 (43.3)	17 (56.7)	0.37**
Heavy	35 (53)	31 (47)	
Fetal sex (N) (%)			
Boy	19 (42.2)	26 (57.8)	0.15**
Girl	29 (56.9)	22 (43.1)	

*Abbreviation*: *GDM* Gestational Diabetes Mellitus, *BMI* Body mass index

^a^ A high school diploma or high school degree is awarded upon high school graduation. An associate degree is an undergraduate degree awarded after a course of post-secondary study lasting two to three years

Values are expressed as means ± SD. *P < *0.05* was considered as significant using Independent t-test for comparison between the two groups. ** *P* < *0.05* was considered as significant using Chi-square test

**Table 2:** The biochemical parameters and dietary intake of the subjects in the control and GDM groups

Variables	Control group	GDM group	*P- value*
FBS (mg/dL)	80.29 ± 1.94	107.83 ± 11.77	< 0.001
HbA1c (%)	4.57 ± 0.46	5.80 ± 0.52	< 0.001
Insulin (μIU/mL)	4.50 ± 1.65	10.63 ± 2.89	< 0.001
HOMA-IR (mmol/L × μIU/mL)	0.89 ± 0.33	2.86 ± 0.97	< 0.001
CRP (mg/L)	4.65 ± 0.99	5.37 ± 0.91	< 0.001
SBP (mmHg)	115.72 ± 3.89	118.87 ± 3.61	< 0.001
DBP (mmHg)	70.08 ± 2.23	71.83 ± 2.27	< 0.001
AEA (ng/ml)	6.12 ± 1.56	9.05 ± 3.66	< 0.001
2-AG (ng/ml)	8.86 ± 2.43	10.76 ± 3.53	0.003
Energy (kcal)	2274.57 ± 238.51	2508.07 ± 166.66	< 0.001
Carbohydrate (gr)	325.49 ± 44.04	367.00 ± 37.75	< 0.001
Protein (gr)	75.71 ± 17.56	80.82 ± 19.59	0.18
Fat (gr)	75.81 ± 13.25	80.13 ± 18.57	0.19

*Abbreviation*: *GDM* Gestational Diabetes Mellitus, *FBG* Fasting blood sugar, *HbA1C* Glycosylated hemoglobin, *HOMA-IR* Hemostatic model assessment-insulin resistance, *CRP* C-reactive protein, *SBP* Systolic blood pressure, *DBP* Diastolic blood pressure, *AEA* Anandamide, *2-AG* 2-Arachidonoylglycerol

Values are expressed as means ± SD. *P* < *0.05* was considered as significant using Independent t-test for comparison between the two groups

**Table 3:** The relationship between endocannabinoids (AEA and 2-AG) with FBS, HbA1c, insulin, HOMA-IR, CRP, SBP, and DBP (dependent variables) in the control group

Variable in the model (AEA)	B^a^	R square	(95% CI)	*P*-value*	Variable in model (2-AG)	B^a^	R square	(95% CI)	*P*-value*
Model 1					Model 1				
FBS (mg/dL)	0.09	0.006	(-0.26- 0.46)	0.59	FBG (mg/dL)	0.02	0.001	(-0.21–0.26)	0.84
HbA1c (%)	0.03	0.01	(-0.05- 0.12)	0.41	HbA1c (%)	-0.01	0.004	(-0.06- 0.04)	0.68
Insulin (μIU/mL)	0.25	0.5	(-0.05- 0.55)	0.10	Insulin (μIU/mL)	0.34	0.25	(0.17- 0.52)	< 0.001
HOMA-IR (mmol/L × μIU/mL)	0.05	0.05	(-0.01- 0.11)	0.09	HOMA-IR (mmol/L × μIU/mL)	0.06	0.25	(0.03- 0.10)	< 0.001
CRP (mg/L)	-0.07	0.01	(-0.26- 0.10)	0.39	CRP (mg/L)	0.07	0.03	(-0.04–0.19)	0.20
SBP (mmHg)	0.29	0.01	(-0.44- 1.02)	0.42	SBP (mmHg)	0.40	0.06	(-0.06–0.86)	0.08
DBP (mmHg)	0.11	0.006	(-0.31- 0.53)	0.59	DBP (mmHg)	0.11	0.01	(-0.15- 0.38)	0.38
Model 2					Model 2				
FBS (mg/dL)	0.08	0.06	(-0.29–0.46)	0.64	FBG (mg/dL)	0.005	0.06	(-0.24- 0.25)	0.96
HbA1c (%)	0.04	0.05	(-0.04–0.13)	0.34	HbA1c (%)	-0.004	0.03	(-0.06- 0.05)	0.90
Insulin (μIU/mL)	0.16	0.24	(-0.12–0.46)	0.25	Insulin (μIU/mL)	0.28	0.37	(0.10- 0.46)	0.002
HOMA-IR (mmol/L × μIU/mL)	0.03	0.24	(-0.02- 0.09)	0.24	HOMA-IR (mmol/L × μIU/mL)	0.05	0.37	(0.02–0.09)	0.002
CRP (mg/L)	-0.08	0.06	(-0.28- 0.10)	0.37	CRP (mg/L)	0.08	0.08	(-0.03- 0.21)	0.16
SBP (mmHg)	0.25	0.16	(-0.46- 0.98)	0.47	SBP (mmHg)	0.37	0.20	(-0.09–0.83)	0.11
DBP (mmHg)	0.15	0.09	(-0.27- 0.58)	0.47	DBP (mmHg)	0.19	0.12	(-0.09- 0.47)	0.17
Model 3					Model 3				
FBS (mg/dL)	0.04	0.14	(-0.32–0.42)	0.79	FBG (mg/dL)	-0.001	0.14	(-0.24- 0.24)	0.99
HbA1c (%)	0.03	0.09	(-0.05–0.13)	0.42	HbA1c (%)	-0.002	0.07	(-0.06- 0.06)	0.95
Insulin (μIU/mL)	0.17	0.28	(-0.12- 0.46)	0.24	Insulin (μIU/mL)	0.27	0.40	(0.09- 0.45)	0.003
HOMA-IR (mmol/L × μIU/mL)	-0.03	0.28	(-0.02- 0.09)	0.27	HOMA-IR (mmol/L × μIU/mL)	0.05	0.39	(0.01- 0.09)	0.004
CRP (mg/L)	-0.06	0.18	(-0.24- 0.12)	0.52	CRP (mg/L)	0.08	0.21	(-0.03- 0.20)	0.17
SBP (mmHg)	0.19	0.23	(-0.51- 0.91)	0.57	SBP (mmHg)	0.35	0.27	(-0.10- 0.81)	
DBP (mmHg)	0.13	0.21	(-0.28- 0.54)	0.53	DBP (mmHg)	0.22	0.25	(-0.04- 0.49)	0.10
Model 4					Model 4				
FBS (mg/dL)	0.04	0.42	(-0.35- 0.43)	0.83	FBG (mg/dL)	-0.10	0.43	(-0.35- 0.14)	0.39
HbA1c (%)	0.009	0.33	(-0.09- 0.11)	0.86	HbA1c (%)	-0.005	0.33	(-0.07- 0.05)	0.86
Insulin (μIU/mL)	0.19	0.50	(-0.12- 0.50)	0.22	Insulin (μIU/mL)	0.26	0.59	(0.08- 0.44)	0.006
HOMA-IR (mmol/L × μIU/mL)	0.03	0.51	(-0.02- 0.09)	0.22	HOMO-IR (mmol/L × μIU/mL)	0.05	0.60	(0.01- 0.08)	0.006
CRP (mg/L)	0.01	0.36	(-0.19- 0.23)	0.86	CRP (mg/L)	0.08	0.39	(-0.04- 0.21)	0.21
SBP (mmHg)	0.49	0.47	(-0.26- 1.24)	0.19	SBP (mmHg)	0.33	0.47	(-0.14- 0.81)	0.16
DBP (mmHg)	0.19	0.36	(-0.27–0.67)	0.40	DBP (mmHg)	0.24	0.40	(-0.04–0.54)	0.09

*Abbreviation*: *GDM* Gestational Diabetes Mellitus, *FBG* Fasting blood sugar, *HbA1C* Glycosylated hemoglobin, *HOMA-IR* Hemostatic model assessment-insulin resistance, *CRP* C-reactive protein, *SBP* Systolic blood pressure, *DBP* Diastolic blood pressure, *AEA* Anandamide, *2-AG* 2-Arachidonoylglycerol

^a^ B coefficient; regression slope

^*^ *P* < 0.05 statistically significant by linear regression. Model 1: unadjusted, model 2: adjusted for energy, carbohydrate, protein, and fat, model 3: adjusted for energy, carbohydrate, protein, and fat, weight gain, and BMI, model 4: adjusted for energy, carbohydrate, protein, and fat, weight gain, BMI, age, education, job, level of physical activity, sex of infant, family history of type 2 diabetes and gestational diabetes, number of pregnancies and weeks of pregnancy

**Table 4:** The relationship between endocannabinoids (AEA and 2-AG) with FBG, HbA1c, insulin, HOMO-IR, CRP, SBP, and DBP (dependent variables) in the GDM group

Variable in model (AEA)	B^a^	R square	(95% CI)	*P*-value*	Variable in model (2-AG)	B^a^	R square	(95% CI)	*P*-value*
Model 1					Model 1				
FBS (mg/dL)	1.27	0.15	(0.4- 2.15)	0.005	FBS (mg/dL)	1.59	0.23	(0.72- 2.46)	0.001
HbA1c (%)	0.04	0.12	(0.01- 0.08)	0.01	HbA1c (%)	0.06	0.18	(0.02- 0.10)	0.003
Insulin (μIU/mL)	0.22	0.08	(0.003- 0.45)	0.04	Insulin (μIU/mL)	0.46	0.31	(0.25- 0.66)	< 0.001
HOMA-IR (mmol/L × μIU/mL)	0.09	0.12	(0.01- 0.16)	0.01	HOMA-IR (mmol/L × μIU/mL)	0.16	0.36	(0.10- 0.23)	< 0.001
CRP (mg/L)	0.03	0.02	(-0.03- 0.11)	0.30	CRP (mg/L)	0.03	0.01	(-0.04- 0.10)	0.41
SBP (mmHg)	0.04	0.002	(-0.24- 0.33)	0.76	SBP (mmHg)	0.10	0.009	(-0.20- 0.40)	0.51
DBP (mmHg)	0.11	0.03	(-0.06- 0.29)	0.20	DBP (mmHg)	0.12	0.04	(-0.05- 0.31)	0.17
Model 2					Model 2				
FBS (mg/dL)	1.17	0.25	(0.30–2.04)	0.009	FBS (mg/dL)	1.54	0.33	(0.68- 2.40)	0.001
HbA1c (%)	0.04	0.44	(0.01- 0.07)	0.008	HbA1c (%)	0.05	0.48	(0.02- 0.08)	0.002
Insulin (μIU/mL)	0.24	0.25	(0.02- 0.45)	0.02	Insulin (μIU/mL)	0.45	0.45	(0.26- 0.64)	< 0.001
HOMA-IR (mmol/L × μIU/mL)	0.09	0.27	(0.02- 0.16)	0.01	HOMA-IR (mmol/L × μIU/mL)	0.16	0.49	(0.10- 0.22)	< 0.001
CRP (mg/L)	0.04	0.25	(-0.02- 0.11)	0.18	CRP (mg/L)	0.04	0.24	(-0.03- 0.11)	0.26
SBP (mmHg)	0.09	0.15	(-0.19–0.37)	0.51	SBP (mmHg)	0.12	0.15	(-0.17- 0.41)	0.41
DBP (mmHg)	0.11	0.28	(-0.05- 0.27)	0.17	DBP (mmHg)	0.15	0.30	(-0.01- 0.32)	0.06
Model 3					Model 3				
FBS (mg/dL)	-0.03	0.44	(-1.05- 0.98)	0. 49	FBS (mg/dL)	0.44	0.45	(-0.64- 1.54)	0.41
HbA1c (%)	0.01	0.49	(-0.02- 0.06)	0.40	HbA1c (%)	0.03	0.51	(-0.01- 0.07)	0.19
Insulin (μIU/mL)	-.02	0.40	(-0.28- 0.23)	0.87	Insulin (μIU/mL)	0.32	0.48	(0.06–0.58)	0.01
HOMA-IR (mmol/L × μIU/mL)	-0.01	0.48	(-0.09- 0.06)	0.76	HOMA-IR (mmol/L × μIU/mL)	0.10	0.55	(0.01- 0.18)	0.01
CRP (mg/L)	0.02	0.26	(-0.06- 0.11)	0.56	CRP (mg/L)	0.01	0.26	(-0.08- 0.11)	0.78
SBP (mmHg)	0.13	0.15	(-0.24, 0.52)	0.47	SBP (mmHg)	0.20	0.16	(-0.21–0.61)	0.33
DBP (mmHg)	0.10	0.34	(-0.1,0.31)	0.32	DBP (mmHg)	0.18	0.37	(-0.04- 0.40)	0.11
Model 4					Model 4				
FBS (mg/dL)	-0.39	0.60	(-1.46- 0.67)	0.45	FBS (mg/dL)	0.45	0.60	(-0.75- 1.66)	0.45
HbA1c (%)	0.01	0.57	(-0.03–0.06)	0.53	HbA1c (%)	0.01	0.57	(-0.03- 0.07)	0.51
Insulin (μIU/mL)	-0.08	0.61	(-0.34–0.17)	0.50	Insulin (μIU/mL)	0.24	0.64	(-0.03–0.53)	0.08
HOMA-IR (mmol/L × μIU/mL)	-0.03	0.64	(-0.12- 0.04)	0.34	HOMA-IR (mmol/L × μIU/mL)	0.08	0.66	(-0.01- 0.17)	0.08
CRP (mg/L)	0.01	0.42	(-0.08- 0.11)	0.74	CRP (mg/L)	0.04	0.43	(-0.06–0.15)	0.41
SBP (mmHg)	0.04	0.47	(-0.33–0.42)	0.80	SBP (mmHg)	0.24	0.49	(-0.17- 0.66)	0.24
DBP (mmHg)	0.04	0.58	(-0.16–0.25)	0.63	DBP (mmHg)	0.25	0.65	(0.03- 0.47)	0.02

*Abbreviation*: *GDM* Gestational Diabetes Mellitus, *FBS* Fasting blood sugar, *HbA1C* Glycosylated hemoglobin, *HOMA-IR* Hemostatic model assessment-insulin resistance, *CRP* C-reactive protein, *SBP* Systolic blood pressure, *DBP* Diastolic blood pressure, *AEA* Anandamide, *2-AG* 2-Arachidonoylglycerol

^a^ B coefficient; regression slope

^*^ *P* < 0.05 statistically significant by linear regression. Model 1: unadjusted, model 2: adjusted for energy, carbohydrate, protein, and fat, model 3: adjusted for energy, carbohydrate, protein, and fat, weight gain, and BMI, model 4: adjusted for energy, carbohydrate, protein, and fat, weight gain, BMI, age, education, job, level of physical activity, sex of infant, family history of type 2 diabetes and gestational diabetes, number of pregnancies and weeks of pregnancy

Odds ratios (95% CI) for gestational diabetes according to endocannabinoid levels (AEA and 2-AG) and anthropometric indices (weight, weight gain, and BMI) were illustrated in Table 5. AEA was significantly associated with an increased risk of GDM in model 1 and model 2 (adjusted for energy, carbohydrate, protein, and fat) and model 3 (adjusted for energy, carbohydrate, protein, and fat, weight gain, and BMI) (OR = 1.22, 95% CI: 1.06–1.41; OR = 1.54, 95% CI: 1.19–1.97; OR = 1.46, 95% CI:1.11–1.91). A positive but not significant association was found for AEA in model 4 (OR = 1.38,95% CI: 0.99–1.92). Similar to AEA, 2-AG was also positively associated with the likelihood of GDM in models 1, 2, and 3 but the association attenuated to null in model 4 (adjusted for energy, carbohydrate, protein, fat, weight gain, BMI, age, education, job, level of physical activity, sex of infant, gestational diabetes, family history of type 2 diabetes, number of pregnancies and weeks of pregnancy) (OR = 1.25; 95% CI: 0.94- 1.65). Weight gain was associated with increased risk of GDM before and after adjustment for confounders (OR = 1.93, 95% CI: 1.35–2.74; OR = 2.07, 95% CI: 1.28–3.33; OR = 1.87, 95% CI: 1.13–3.12; OR = 3.24, 95% CI: 1.55–6.8).

**Table 5:** Odds ratios (95% CI) for gestational diabetes according to endocannabinoids (AEA and 2-AG) and anthropometric indices (weight, weight gain, and BMI)

Variable	Or (CI)	B^a^	*P- value
AEA (ng/ml)			
Model 1	1.22 (1.06–1.41)	0.20	0.004
Model 2	1.54 (1.19–1.97)	0.43	0.001
Model 3	1.46 (1.11–1.91)	0.37	0.006
Model 4	1.38 (0.99–1.92)	0.32	0.05
2-AG (ng/ml)			
Model 1	1.22 (1.06–1.41)	0.20	0.004
Model 2	1.33 (1.10–1.61)	0.28	0.003
Model 3	1.23 (1.007–1.50)	0.20	0.04
Model 4	1.25 (0.94–1.65)	0.22	0.11
Weight (kg)			
Model 1	1.14 (1.04–1.26)	0.13	0.006
Model 2	1.11 (0.99–1.23)	0.10	0.05
Model 3	1.05 (0.92–1.20)	0.05	0.40
Model 4	1.003 (0.85–1.17)	0.003	0.97
Weight gain (kg)			
Model 1	1.93 (1.35- 2.74)	0.65	< 0.001
Model 2	2.07 (1.28–3.33)	0.72	0.003
Model 3	1.87 (1.12- 3.12)	0.62	0.01
Model 4	3.24 (1.55–6.80)	1.17	0.002
BMI (kg/m^2^)			
Model 1	2.06 (1.38- 3.06)	0.72	< 0.001
Model 2	1.59 (1.04–2.43)	0.46	0.02
Model 3	1.54 (0.77- 3.05)	0.43	0.21
Model 4	2.18 (0.88- 5.42)	0.78	0.09

*Abbreviation*: *AEA* Anandamide, *2-AG* 2-Arachidonoylglycerol, *BMI* Body mass index

^a^ B coefficient; regression slope

^*^ *P* < *0.05* statistically significant by Multivariable logistic regression. Model 1: unadjusted, model 2: adjusted for energy, carbohydrate, protein, and fat, model 3: adjusted for energy, carbohydrate, protein, and fat, weight gain, and BMI, model 4: adjusted for energy, carbohydrate, protein, and fat, weight gain, BMI, age, education, job, level of physical activity, sex of infant, family history of type 2 diabetes and gestational diabetes, number of pregnancies and weeks of pregnancy

## Discussion

For the first time, our findings demonstrated that blood levels of ECs were significantly higher in pregnant women with GDM compared to the control group. Also, ECs levels were associated with the likelihood of GDM independent of BMI and weight gain. Plasma levels of 2-AG were also significantly associated with insulin levels and HOMA-IR in the control group. In fact, some components of ECS, in particular NAPE-PLD (N-acyl-phosphatidylethanolamine [NAPE]-selective phospholipase D), fatty acid amide hydrolase (FAAH), and CB receptors might also be modulated during the oocyte transportation from the ovary to the implantation site. In other words, it has been widely suggested that the endogenous levels of AEA are finely and tightly regulated from the very beginning of pregnancy and any dysregulation of this parameter severely compromise the pregnancy outcome [24].

Previous studies also found increased circulating levels of AEA and 2-AG in obese women [10, 25–27], obese men [28], patients with nonalcoholic fatty liver disease (NAFLD) [29], hyperglycemia, and T2DM patients [15].

Osei-Hyiaman et al. showed elevated blood concentrations of AEA and 2-AG in women with obesity [25]. In the present study, the blood levels of AEA and 2-AG were higher than those reported by cote et al. [28]. It is suggested that elevated endocannabinoid concentrations may be secondary to marked downregulation of FAAH gene expression in adipose tissue of obese women [25]. Furthermore, Sipe et al., suggested that dysregulation/upregulation of the ECS in obesity may be related to genetic predisposition such as the FAAH 385 A/A missense polymorphism [30].

On the other hand, Abdulnour et al., for the first time, showed that circulating levels of the 2-AG are higher in insulin-resistant obese individuals compared to insulin-sensitive obese postmenopausal women [9]. It has been previously indicated that an increase in 2-AG levels is associated with insulin resistance in adipose tissue. Overactivation of CB1 receptor by elevated 2-AG local tissue levels is proposed to decrease glucose uptake in skeletal muscle, raise abdominal adiposity and free fatty acid flow from adipose tissue to the liver developing the risk of insulin resistance [31, 32]. Also, CB1 receptor overactivation can prevent the translocation of glucose transporter type 4 (GLUT4), and has an adverse impact on genes controlling the insulin sensitivity in skeletal muscles [33, 34]. Additionally, upregulation of the ECS can lead to beta-cell loss via stimulation of the Nlrp3-ASC inflammasome in infiltrating macrophages [35].

In agreement with our findings, some studies have also reported 2-AG as the most efficacious endocannabinoid relating to dyslipidemia, visceral adiposity, and insulin resistance [9, 36]. In a study by Cote et al., the blood concentration of 2-AG, but not AEA, had a positive significant relation with waist circumference, BMI, insulin levels, triglyceride, and negative relation with high-density lipoprotein -cholesterol (HDL-c) and adiponectin [28].

It has been revealed that 2-AG can act as an insulin resistance biomarker in postmenopausal women and could be used to discriminate the insulin-sensitive obese from insulin-resistant obese phenotypes [9]. In hyperglycemic conditions such as obesity, prediabetes, and type 2 diabetes the endocannabinoid system is dysregulated in β-cells. The overstimulation of CB1 receptors might reinforce the insulin release which leads to permanent hyperinsulinemia. This might start a vicious circle with further elevation of endocannabinoid levels in β-cells which in turn triggers the adipocyte hypertrophy and endocannabinoid hyperactivity in adipocytes, and subsequent increase in lipid levels, decrease in adiponectin levels [37].

Moreover, circulating levels of ECs are associated with age, anthropometric and metabolic parameters. In a study by Fanelli et al. age was found to positively influence ECs levels in female particpants aged between 35–52 yrs [38]. Whereas in the present study age range of the study population was 25–37 yrs.

Several studies have targeted the ECS to promote metabolic health. For example, treatment with rimonabant (a CB1 receptor antagonist) induced weight loss and improved dysregulations in lipid and glucose metabolism in mice fed a high-fat diet and obese individuals [39–43]. White adipocytes express functional CB1 receptors which levels are higher in obese rats and their blockade leads to increased levels of adiponectin [15, 44].

Additionally, dietary interventions can also reduce the ECs levels. Regarding dietary long-chain polyunsaturated fatty acids, they could decrease plasma ECs levels, inflammatory mediators, and ectopic fat deposition by decreasing the availability of ECs biosynthetic precursors [45–47].

However, the effect of weight loss stemmed from hypocaloric diets on ECs status is variable. It was shown that at least 10% weight loss was needed to affect the circulating concentration of 2-AG and AEA [10, 36]. Recently, the effects of simultaneous weight loss diet and 30-g whey protein supplementation for 8 weeks on 2-AG and anandamide were observed in obese women [10, 21].

A key strength of the present study was the assessment of ECs levels in women with GDM for the first time. However, these findings are limited by the use of the ELISA kits in determining of the blood ECs instead of HPLC. Also, ECs metabolizing enzymes, Corticotropin-Releasing Hormone (CRH) levels, and behavioral/psychological status were not evaluated. The ECS is a vital neuromodulatory system associated with several psychiatric, neurodegenerative, and motor disorders [48].

## Conclusions

Our findings showed that blood levels of ECs were significantly higher in pregnant women with GDM compared to the control group for the first time. Also, ECs levels were associated with the likelihood of GDM independent of BMI and weight gain. This research has provided additional evidence with respect to the role of ECs in the pathogenesis of GDM.

## Data Availability

The datasets used and/or analyzed during the current study are available from the corresponding author on reasonable request.

## References

[1] Wang H, Li N, Chivese T, Werfalli M, Sun H, Yuen L (2022). IDF diabetes atlas: estimation of global and regional gestational diabetes mellitus prevalence for 2021 by International Association of Diabetes in Pregnancy Study Group’s Criteria. Diabetes Res Clin Pract.

[2] Gyasi-Antwi P, Walker L, Moody C, Okyere S, Salt K, Anang L, et al. Global prevalence of gestational diabetes mellitus: a systematic review and meta-analysis. New American Journal of Medicine. 2020;1(3):1–10.

[3] Lee KW, Ching SM, Ramachandran V, Yee A, Hoo FK, Chia YC (2018). Prevalence and risk factors of gestational diabetes mellitus in Asia: a systematic review and meta-analysis. BMC Pregnancy Childbirth.

[4] Jafari-Shobeiri M, Ghojazadeh M, Azami-Aghdash S, Naghavi-Behzad M, Piri R, Pourali-Akbar Y (2015). Prevalence and risk factors of gestational diabetes in Iran: a systematic review and meta-analysis. Iran J Public Health.

[5] Borissova A-M, Trifonova B, Dakovska L, Michaylova E, Vukov M (2021). Age, obesity, family history, previous gestational diabetes are major risk factors for hyperglycemia in pregnant bulgarian women. Eur J Prev Cardiol.

[6] Casagrande SS, Linder B, Cowie CC (2018). Prevalence of gestational diabetes and subsequent type 2 diabetes among US women. Diabetes Res Clin Pract.

[7] Bianchi C, Battini L, Aragona M, Lencioni C, Ottanelli S, Romano M (2017). Prescribing exercise for prevention and treatment of gestational diabetes: review of suggested recommendations. Gynecol Endocrinol.

[8] Kramer CK, Campbell S, Retnakaran R (2019). Gestational diabetes and the risk of cardiovascular disease in women: a systematic review and meta-analysis. Diabetologia.

[9] Abdulnour J, Yasari S, Rabasa-Lhoret R, Faraj M, Petrosino S, Piscitelli F (2014). Circulating endocannabinoids in insulin sensitive vs. insulin resistant obese postmenopausal women. a MONET group study. Obesity..

[10] Haidari F, Aghamohammadi V, Mohammadshahi M, Ahmadi-Angali K, Asghari-Jafarabadi M (2020). Whey protein supplementation reducing fasting levels of anandamide and 2-AG without weight loss in pre-menopausal women with obesity on a weight-loss diet. Trials.

[11] Watkins BA, Kim J (2015). The endocannabinoid system: directing eating behavior and macronutrient metabolism. Front Psychol.

[12] Horváth B, Mukhopadhyay P, Haskó G, Pacher P (2012). The endocannabinoid system and plant-derived cannabinoids in diabetes and diabetic complications. Am J Pathol.

[13] Silvestri C, Di Marzo V (2013). The endocannabinoid system in energy homeostasis and the etiopathology of metabolic disorders. Cell Metab.

[14] Bluher M, Engeli S, Kloting N, Berndt J, Fasshauer M, Bátkai S (2006). Dysregulation of the peripheral and adipose tissue endocannabinoid system in human abdominal obesity. Diabetes.

[15] Matias I, Gonthier M-P, Orlando P, Martiadis V, De Petrocellis L, Cervino C (2006). Regulation, function, and dysregulation of endocannabinoids in models of adipose and β-pancreatic cells and in obesity and hyperglycemia. J Clin Endocrinol Metab.

[16] Harlev A, Wiznitzer A (2010). New insights on glucose pathophysiology in gestational diabetes and insulin resistance. Curr DiabRep.

[17] van Eyk HJ, van Schinkel LD, Kantae V, Dronkers CE, Westenberg JJ, de Roos A (2018). Caloric restriction lowers endocannabinoid tonus and improves cardiac function in type 2 diabetes. Nutr Diabetes.

[18] Mellitus AGD (2004). Position statement. Diabetes Care.

[19] Metzger BE, Buchanan TA, Coustan DR, De Leiva A, Dunger DB, Hadden DR (2007). Summary and recommendations of the fifth international workshop-conference on gestational diabetes mellitus. Diabetes care..

[20] Craig C, Marshall A, Sjostrom M, Bauman A, Lee P, Macfarlane D (2017). International physical activity questionnaire-short form. J Am Coll Health.

[21] Haidari F, Aghamohammadi V, Mohammadshahi M, Ahmadi-Angali K (2017). Effect of whey protein supplementation on levels of endocannabinoids and some of metabolic risk factors in obese women on a weight-loss diet: a study protocol for a randomized controlled trial. Nutr J.

[22] Little RR, Roberts WL (2009). A review of variant hemoglobins interfering with hemoglobin A1c measurement. J Diabetes Sci Technol.

[23] Shen Y, Prinyawiwatkul W, Xu Z (2019). Insulin: a review of analytical methods. Analyst.

[24] Battista N, Pasquariello N, Di Tommaso M, Maccarrone M (2008). Interplay between endocannabinoids, steroids and cytokines in the control of human reproduction. J Neuroendocrinol.

[25] Osei-Hyiaman D, DePetrillo M, Pacher P, Liu J, Radaeva S, Bátkai S (2005). Endocannabinoid activation at hepatic CB 1 receptors stimulates fatty acid synthesis and contributes to diet-induced obesity. J Clin Investig.

[26] Yagin NL, Aliasgari F, Alizadeh M, Aliasgharzadeh S, Mahdavi R (2020). Comparison of endocannabinoids levels, FAAH gene polymorphisms, and appetite regulatory substances in women with and without binge eating disorder: a cross-sectional study. Nutr Res.

[27] Yagin NL, Aliasgharzadeh S, Alizadeh M, Aliasgari F, Mahdavi R (2020). The association of circulating endocannabinoids with appetite regulatory substances in obese women. Obes Res Clin Pract.

[28] Cote M, Matias I, Lemieux I, Petrosino S, Almeras N, Despres J (2007). Circulating endocannabinoid levels, abdominal adiposity and related cardiometabolic risk factors in obese men. Int J Obes.

[29] Zelber-Sagi S, Azar S, Nemirovski A, Webb M, Halpern Z, Shibolet O (2017). Serum levels of endocannabinoids are independently associated with nonalcoholic fatty liver disease. Obesity.

[30] Sipe J, Waalen J, Gerber A, Beutler E (2005). Overweight and obesity associated with a missense polymorphism in fatty acid amide hydrolase (FAAH). Int J Obes.

[31] Ginsberg HN, Woods SC (2009). The endocannabinoid system: potential for reducing cardiometabolic risk. Obesity (Silver Spring, Md).

[32] Saavedra L (2007). Endocannabinoid system and cardiometabolic risk. Clin Pharmacol Ther.

[33] Heyman E, Gamelin FX, Aucouturier J, Di Marzo V (2012). The role of the endocannabinoid system in skeletal muscle and metabolic adaptations to exercise: potential implications for the treatment of obesity. Obes Rev..

[34] Lindborg K, Teachey M, Jacob S, Henriksen E (2010). Effects of in vitro antagonism of endocannabinoid-1 receptors on the glucose transport system in normal and insulin-resistant rat skeletal muscle. Diabetes Obes Metab.

[35] Jourdan T, Godlewski G, Cinar R, Bertola A, Szanda G, Liu J (2013). Activation of the Nlrp3 inflammasome in infiltrating macrophages by endocannabinoids mediates beta cell loss in type 2 diabetes. Nat Med.

[36] Di Marzo V, Cote M, Matias I, Lemieux I, Arsenault B, Cartier A (2009). Changes in plasma endocannabinoid levels in viscerally obese men following a 1 year lifestyle modification programme and waist circumference reduction: associations with changes in metabolic risk factors. Diabetologia.

[37] Juan-Picó P, Fuentes E, Bermúdez-Silva FJ, Díaz-Molina FJ, Ripoll C, de Fonseca FR (2006). Cannabinoid receptors regulate Ca2+ signals and insulin secretion in pancreatic β-cell. Cell Calcium.

[38] Fanelli F, Di Lallo VD, Belluomo I, De Iasio R, Baccini M, Casadio E (2012). Estimation of reference intervals of five endocannabinoids and endocannabinoid related compounds in human plasma by two dimensional-LC/MS/MS. J Lipid Res.

[39] Ravinet Trillou C, Arnone M, Delgorge C, Gonalons N, Keane P, Maffrand J-P (2003). Anti-obesity effect of SR141716, a CB1 receptor antagonist, in diet-induced obese mice. American Am J Physiol Regul Integr Comp Physiol.

[40] Gary-Bobo M, Elachouri G, Gallas JF, Janiak P, Marini P, Ravinet-Trillou C (2007). Rimonabant reduces obesity-associated hepatic steatosis and features of metabolic syndrome in obese Zucker fa/fa rats. Hepatology.

[41] Van Gaal LF, Rissanen AM, Scheen AJ, Ziegler O, Rössner S, Group R-ES (2005). Effects of the cannabinoid-1 receptor blocker rimonabant on weight reduction and cardiovascular risk factors in overweight patients: 1-year experience from the RIO-Europe study. Lancet..

[42] Bergholm R, Sevastianova K, Santos A, Kotronen A, Urjansson M, Hakkarainen A (2013). CB 1 blockade-induced weight loss over 48 weeks decreases liver fat in proportion to weight loss in humans. Int J Obes.

[43] Lazzari P, Sanna A, Mastinu A, Cabasino S, Manca I, Pani L (2011). Weight loss induced by rimonabant is associated with an altered leptin expression and hypothalamic leptin signaling in diet-induced obese mice. Behav Brain Res.

[44] Bensaid M, Gary-Bobo M, Esclangon A, Maffrand J, Le Fur G, Oury-Donat F (2003). The cannabinoid CB1 receptor antagonist SR141716 increases Acrp30 mRNA expression in adipose tissue of obese fa/fa rats and in cultured adipocyte cells. Mol Pharmacol.

[45] Banni S, Carta G, Murru E, Cordeddu L, Giordano E, Sirigu AR (2011). Krill oil significantly decreases 2-arachidonoylglycerol plasma levels in obese subjects. Nutr Metab.

[46] Matias I, Carta G, Murru E, Petrosino S, Banni S, Di Marzo V (2008). Effect of polyunsaturated fatty acids on endocannabinoid and N-acyl-ethanolamine levels in mouse adipocytes. Biochim Biophys Acta..

[47] Batetta B, Griinari M, Carta G, Murru E, Ligresti A, Cordeddu L (2009). Endocannabinoids may mediate the ability of (n-3) fatty acids to reduce ectopic fat and inflammatory mediators in obese Zucker rats. J Nutr.

[48] Bahji A, Breward N, Duff W, Absher N, Patten SB, Alcorn J (2022). Cannabinoids in the management of behavioral, psychological, and motor symptoms of neurocognitive disorders: a mixed studies systematic review. J Cannabis Res.

